# Structure and dynamics of the operon map of *Buchnera aphidicola *sp. strain APS

**DOI:** 10.1186/1471-2164-11-666

**Published:** 2010-11-25

**Authors:** Lilia Brinza, Federica Calevro, Gabrielle Duport, Karen Gaget, Christian Gautier, Hubert Charles

**Affiliations:** 1 INSA-Lyon, UMR203 BF2I, INRA, Biologie Fonctionnelle Insectes et Interactions, Bât. Louis Pasteur 20 ave. Albert Einstein, F-69621 Villeurbanne, France; 2 Université de Lyon, Univ Lyon 1, CNRS UMR5557 Ecologie Microbienne, INRA, F-69622 Villeurbanne, France; 3 Université de Lyon, INRIA Bamboo, F-69621 France

## Abstract

**Background:**

Gene expression regulation is still poorly documented in bacteria with highly reduced genomes. Understanding the evolution and mechanisms underlying the regulation of gene transcription in *Buchnera aphidicola*, the primary endosymbiont of aphids, is expected both to enhance our understanding of this nutritionally based association and to provide an intriguing case-study of the evolution of gene expression regulation in a reduced bacterial genome.

**Results:**

A Bayesian predictor was defined to infer the *B. aphidicola *transcription units, which were further validated using transcriptomic data and RT-PCR experiments. The characteristics of *B. aphidicola *predicted transcription units (TUs) were analyzed in order to evaluate the impact of operon map organization on the regulation of gene transcription.

On average, *B. aphidicola *TUs contain more genes than those of *E. coli*. The global layout of *B. aphidicola *operon map was mainly shaped by the big reduction and the rearrangements events, which occurred at the early stage of the symbiosis. Our analysis suggests that this operon map may evolve further only by small reorganizations around the frontiers of *B. aphidicola *TUs, through promoter and/or terminator sequence modifications and/or by pseudogenization events. We also found that the need for specific transcription regulation exerts some pressure on gene conservation, but not on gene assembling in the operon map in *Buchnera*. Our analysis of the TUs spacing pointed out that a selection pressure is maintained on the length of the intergenic regions between divergent adjacent gene pairs.

**Conclusions:**

*B. aphidicola *can seemingly only evolve towards a more polycistronic operon map. This implies that gene transcription regulation is probably subject to weak selection pressure in *Buchnera *conserving operons composed of genes with unrelated functions.

## Background

Understanding the regulation of gene expression necessitates deciphering how organisms sense their environment and respond to it. The topic is even more crucial when looking at tight symbiotic interactions within which partners maintain biochemical relationships shaping in a drastic way their genomes. Our study is focused on *Buchnera aphidicola*, the primary endosymbiont of aphids, and one of the most studied obligate intracellular bacteria of insects, and analyzes the evolution of the regulation of gene expression in the context of intracellular symbiosis.

In prokaryotes, gene expression and gene regulation are governed by diversified and highly evolving mechanisms [1]. In the free-living bacteria so far studied, genes are under the combined control of several mechanisms which can be enumerated following the flow of information from the gene structure to protein function: the gene copy number, the susceptibility of a gene to be transcribed (governed by the initiation transcription rate, the elongation and termination efficiencies), the composition of the transcription units (TUs), the stability and the degradation of the mRNA, the efficiency with which the mRNA is translated (mainly the codon usage), and the effect of post-translational modifications. Prokaryotic TUs may contain either one (monocistronic) or several (polycistronic) distinct juxtaposed genes, all controlled by a common regulatory region and transcribed into one long mRNA molecule. Finally, more general regulation mechanisms, such as chromosome topology (*e.g*., chromosome supercoiling), have also been reported to control global gene transcription physically [2].

Until 10 years ago, it was thought that the control of expression at the transcription initiation level was the dominant form of regulation in prokaryotes, for obvious reasons of efficiency and economy [1]. This assumption seemed natural given the apparent simplicity of prokaryotic transcriptomes (the small proportion of non-coding sequences, and lack of introns and alternative splicing). However, very recently, new technologies have highlighted the complexity and dynamic nature of gene regulation in prokaryotes (*e.g*., detection of sRNA, modulation of operon structures, antisense transcription) [3].

Our bacterial model, *B. aphidicola*, has a close phylogenetic relationship with the large-genome free-living bacterium *Escherichia coli*; the *B. aphidicola *genome is essentially an *E. coli *subset [4]. Since its association with aphids began, about 150-200 MY ago [5,6], *B. aphidicola *has undergone major genomic modifications as a result of its intracellular lifestyle: a major AT bias (70% of the genome consists of AT bases); genome shrinkage (*B. aphidicola *sequenced genomes range from 420 kb to 650 kb, while the last common ancestor shared with *E. coli *had approximately 2 Mb); a rapid evolutionary rate and lack of recombination [7-9].

Genomic analysis of *B. aphidicola *from the pea aphid, *Acyrthosiphon pisum *(BAp), has revealed the absence of genes encoding for the regulatory systems usually found in *E. coli *and Proteobacteria [10]. Indeed, two-component regulatory systems are absent. In addition, only two sigma factors are present in BAp: *rpoD *and *rpoH*. None of the orthologous regulators of the operons that encode the enzymes of the essential amino acids pathways in *E. coli *are present in *B. aphidicola *[10]. The genes of *B. aphidicola *have no leader sequences, and the bacterium does not use attenuation systems [10]. The genes encoding adenylate cyclase (*cyaA*) and the AMPc receptor (*crp*) are also absent in BAp, indicating that it can survive only on a glucose carbon source [10]. However, there is experimental evidence of gene expression regulation in *Buchnera*, since nutritional osmotic stress or organ and embryonic stage specificities are associated with differing gene expression profiles [11,12].

It is difficult to address the question of gene regulation in *B. aphidicola*, because of the impossibility of direct experimentation on this un-culturable bacterium. Promoter identification also seems to be compromised by the strong AT-bias of the intergenic regions. In this context, we decided to study the TUs of *B. aphidicola*, and the first part of our work was devoted to predicting the TUs of *B. aphidicola *and its operon map (*i.e*., the overall polycistronic TU layout as defined by Edwards [13]).

The operon map is a highly dynamic structure, its evolution is governed locally by sequence evolution (base mutations), and more generally by genome rearrangements (*e.g. *inversions, transpositions, deletions, insertions and duplications). Both processes affect not only the genomic elements involved in gene expression regulation, such as promoters and terminators, but also the overall organization of these elements on the chromosome (gene order, proximity, strand, etc.).

The methods predicting the composition of TUs (*i.e. *the genes each TU contains) are basically classifiers (supervised or unsupervised) assigning the pairs of adjacent genes to either the intra-TU class, or the inter-TU class. Most frequently, statistical approaches are used to develop these classifiers. Several features of the adjacent gene pairs, such as the intergenic distance, the conservation of gene pairs across multiple genomes, the functional similarity between adjacent genes, the involvement of a given gene pair in the same biological pathway or physical complex (e.g., protein-protein interaction), gene expression correlations and some other features are used in the classification. Some of these methods have been applied to all sequenced and annotated bacterial genomes, and their predicted collections of TUs are stored in dedicated or general microbial databases. We found the set of BAp TUs in three databases: BioCyc [14], DOOR [15] and MicrobesOnline [16].

The TU prediction method used in the BioCyc database [17] uses intergenic distance, functional similarity, metabolic network information and protein-protein network information to construct a log-likelihood table, which was used to make TU predictions. The method was trained on the *E. coli *data set. The DOOR method [18] uses a data-mining classifier trained on *E. coli *and *Bacillus subtilis*, including the intergenic distance, neighborhood conservation across multiple genomes, phylogenetic distance between adjacent genes, information from short DNA motifs, similarity score between GO terms of gene pairs and the length ratio between a pair of genes. Finally, MicrobesOnline [19] predictions were made by an unsupervised approach using intergenic distance, neighborhood conservation, functional similarity and the similarity of the gene codon adaptation index (CAI). As these three predictions were not especially designed for *B. aphidicola *and only partially overlap (see the Results section), we proposed a new set of TUs for *B. aphidicola*, based on a Bayesian prediction, that we compared with the three already available in the literature, and further validated experimentally.

In the second part of this study, we investigated the characteristics of *B. aphidicola *TUs, and evaluated the impact of operon map organization on the regulation of gene transcription. We also tested the influence of TU organization on coding sequence length dynamics during the evolution of the *B. aphidicola *lineage. Understanding the mechanisms of gene expression regulation in *B. aphidicola *is of interest for two reasons: first, it would provide a better understanding of the nutritionally based aphid - *B. aphidicola *association, and second, it provides an intriguing case study of the evolution of gene regulation in a reduced bacterial genome.

## Methods

### Data organization and training data set

For training our TU finder, a data set was constructed using genomic and transcription unit information for *E. coli *from the Regulon database [20,21]. Using the nomenclature of Salgado et al. [22], we defined several classes of pairs of adjacent genes as follows (summarized in Additional File 1):

- if both genes of the pair belong to an experimentally identified transcription unit, the pair class was designated "Same TU" (STU),

- if only one of the genes of the pair belong to an experimentally determined TU, or if the genes have opposite transcription directions, then the pair class was designated "Different TU" (DTU);

- if both genes belong to a TU exclusively predicted by computational methods, the pair class was designated "Not Known" (NK).

In RegulonDB, some overlapping polycistronic TUs can be found, due to the presence of alternative promoters or alternative terminators. A pair of same-strand adjacent genes was classified "STU", if there was at least one polycistronic TU containing this pair.

NK pairs were excluded from the training data set, as well as the operon leader peptides. The polycistronic TUs carrying leader peptides are not structurally representative of the polycistronic TU population in *B. aphidicola*, because they are normally involved in transcription attenuation, a regulatory mechanism absent in *B. aphidicola *[10]. In this way, we constructed a training data set as similar as possible to the *B. aphidicola *model.

### Features, feature selection and predictor construction

#### Intergenic distance

The intergenic distance is the number of base pairs separating two adjacent coding sequences, for overlapping genes the intergenic distance is negative. This feature has been shown to be critical in operon prediction [22], and to be the best single-feature predictor of *E. coli *operons [23]. Like Salgado et al. [22] and Romero et al. [17], we categorized the intergenic distance in 10-bp intervals.

#### Transcription Rho-independent terminators

Rho-independent terminators are genomic elements which transient structure induces the stop of transcription by creating a loop within the mRNA extremity during the elongation step. Their sequence consists of a GC-rich hairpin followed by a thymine residue enriched sequence [24]. For both *E. coli *and *B. aphidicola*, we used the terminators predicted by TransTermHP [25].

#### Configurations tested for the Bayesian predictor DisTer

We tested three models for our Bayesian TUs predictor (DisTer). The features included in each model, and the formula used to calculate the probability that a pair of adjacent genes would be classified as STU are as follows:

(1) the intergenic distance and the presence of the terminator, assuming that these properties are independent;

(2) the joint distribution of the intergenic distance and the presence of the terminator;

(3) the joint distribution of the intergenic distance and the TransTermHP score of the terminator.

**Figure 1:**
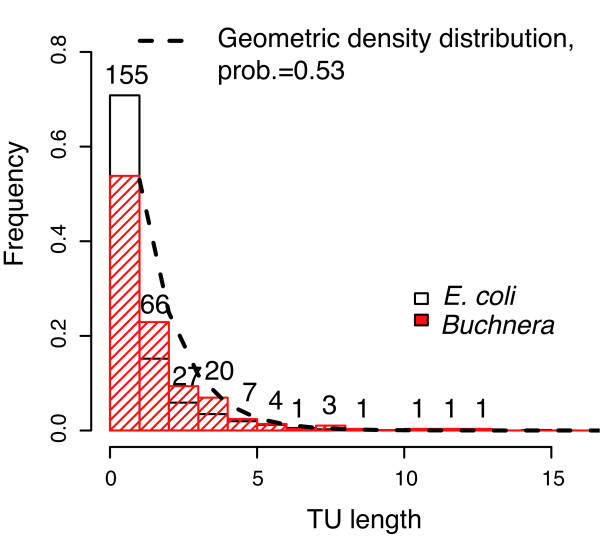
**Distributions of the TU length (number of genes) in *E. coli *and *Buchnera***. The two distributions are significantly different. The numbers on the bars indicate the number of TUs of that length in *Buchnera*. The dotted line shows the estimated TUs length distribution with a geometric distribution.

The corresponding formulae are presented in the Additional File 2. To estimate the prior probability that a pair of adjacent genes would belong to the same TU, we assumed that the number of genes in a TU follows a geometric distribution, *P*(*L*_TU _= *n*) = *P*(STU pair)^n-1 ^(1 - *P*(STU pair)). This is the simplest statistical model. The expected value of the distribution is 1/(1 - *P*(STU pair)), so we calculated the *P*(STU pair) using the mean of *L*_*TU*_, (${\bar{L}}_{TU}$): *P*(STU pair) = $\frac{{\bar{L}}_{TU}-1}{{\bar{L}}_{TU}}=0.53$ (the density curve is presented on Figure 1).

**Figure 2:**
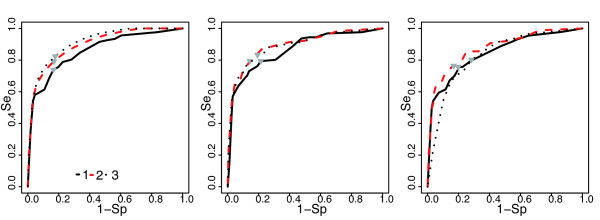
**ROC curves of the three prediction models tested for DisTer**. ROC curves (showing 1-Sp, where Sp is the proportion of true DTU pairs correctly classified as DTU by our method, as a function of Se, the proportion of true STU pairs correctly classified as STU by our method) are evaluating the resubstitution error rate (left) and the model performance when the model is trained on 80% of the *E. coli *data and tested on the other 20% (middle), or using the leave-one-out technique (right). The red lines correspond to the second predictor model (DisTer), which was chosen for *Buchnera *operon map prediction. The grey arrows indicate the nearest point of the curves to the left upper corner. For the description of the models 1, 2 and 3 see the Methods section.

The class attributed is normally that with the greatest estimated probability, and in our case, a gene pair could thus have been classified as an STU when the *p*(STU|pair properties) was >0.5, and as a DTU if it was ≤0.5. However, instead of doing this, we looked for the most discriminating value for the probability threshold, regarding the model accuracy and its predictive value. Probability threshold values between 0.05 and 1 were tested using the entire training set in order to evaluate the training data error rate, also known as the resubstitution error rate (here the same data set is used both to train the predictor and to assess its performance). This error rate is a good indicator of the uncertainty of the classification rules (Figure 2, left and Additional File 3). We used the sensitivity (the proportion of true STU pairs correctly classified as STUs by our method) and the specificity (the proportion of true DTUs correctly classified as DTU by our method) in order to evaluate the quality of the predictions.

The predictive value of the model was also tested for the same probability threshold values using the holdout method: the *E. coli *data set was split into training (80%) and test (20%) data sets (Figure 2, middle), but also by a leave-one-out cross validation method (Figure 2, right). This last is an iterative approach in which each gene pair in the training set of *N *gene pairs is left out during one iteration. The model is trained with the remaining *N-1 *gene pairs, and is then used to classify the gene pair left out. We used the integral prediction made in this way (*N *iterations) to evaluate the performance of our model.

On the Figure 2 the nearest point to the left corner of one curve (indicated by the arrow) is the point that minimizes the sum of the squares of the proportions of false predictions. For each model we considered that the probability threshold corresponding to this point on the curve gave the best performances. The best probability threshold value for the resubstitution error rate and the best probability threshold value for the model performances were not the same except for the second model (the probability threshold values can be visualized on Additional File 3). It is interesting to note that the second model predictor produced the best performances except when the entire *E. coli *data set was used, which was to be expected, since the third model divides the data into more classes than the second one. Hence the second model has the best predictive capability. Finally the second model (using joint distribution of the intergenic distance and terminator presence) with a 0.5 threshold, represents a good compromise between the resubstitution error rate (Se = 79% and Sp = 83%) and performance (Se = 78% and Sp = 84%), and was therefore the one chosen to predict the *B. aphidicola *TUs. All the calculations were performed using R 2.6.1 software [26].

### Coding sequence length comparison

For each pair of *Buchnera*-*E. coli *orthologues, we traced the dynamics of the evolution of the sequence length in both lineages using *Vibrio cholerae *(accession number AE003852 and AE003853) as an outgroup. *Pseudomonas aeruginosa *(CP000438) or *Haemophilus influenzae *(L42023) were used when no orthologue could be found in *V. cholerae*. As no gene duplication has occurred in the *B. aphidicola *lineage since its divergence from *E. coli *[4], we used the bidirectional best hit method to identify the orthologues of *B. aphidicola *genes. Comparing the length of *B. aphidicola *genes to those of their *E. coli *orthologues (Δ_L_E.coli-L_Bu_) and of the outgroup orthologue lengths (Δ_L_Ext-L_Bu_), we defined six classes:

(1) genes with the same length in *B. aphidicola *and *E. coli *(Δ_L_E. coli-L_Bu _= 0); (2) genes which length increased in the *E. coli *lineage (if |Δ_L_Ext-L_Bu_| < |Δ_L_E. coli-L_Ext_| and Δ_L_E. coli-L_Bu _> 0); (3) genes which length decreased in the *E. coli *lineage (if |Δ_L_Ext-L_Bu_| < |Δ_L_E. coli-L_Ext_| and Δ_L_E. coli-L_Bu _< 0); (4) genes which length increased in the *B. aphidicola *lineage (if |Δ_L_Ext-L_Bu_| > |Δ_L_E. coli-L_Ext_| and Δ_L_E. coli-L_Bu _> 0); (5) genes which length decreased in the *B. aphidicola *lineage (if |Δ_L_Ext-L_Bu_| > |Δ_L_E. coli-L_Ext_| and Δ_L_E. coli-L_Bu _< 0); (6) genes for which the lineage undergoing a length change could not be identified (if |Δ_L_Ext-L_Bu_| = |Δ_L_E. coli-L_Ext_| and Δ_L_E. coli-L_Bu _≠ 0).

### Experimental validation of *Buchnera *TUs

*B. aphidicola *TUs were experimentally validated using the protocol devised by Charaniya et al. [27].

*B. aphidicola *cells were purified from about 900 mg of aphids, using the procedure described in Charles et al. [28]. Total gDNA was extracted using the QIAamp DNA Mini Kit (Qiagen, Helden, Germany). The gDNA was used for tuning the PCR conditions, and as positive control for the RT-PCR reactions. Total RNA was isolated and purified with RNeasy kit (Qiagen) as described by Calevro et al. [29]. Purity and RNA integrity were evaluated by NanoDrop^® ^determination of the absorbance at 230 nm, 260 nm and 280 nm, and by denaturing agarose gel electrophoresis respectively (data not shown). The total RNA was then treated with Turbo DNA-free™ DNase (Ambion, Austin, TX, USA). The reverse transcription was performed from 1 μg RNA, using random hexamers and SuperScript™ III, according to the SuperScript™ First-Strand Synthesis system kit for the RT-PCR kit protocol (Invitrogen, Paisley, UK). The addition of 1 μl RNase H at the end of the reverse transcription, combined with incubation for 20 minutes at 37°C, eliminated all the RNA initially present in the solution. Two microliters from the reverse transcription reaction were used for each of the subsequent RT-PCR reactions. Specific oligo primers were designed for each product (gene pair) with the Oligo 6 software (Molecular Biology Insight, Inc). cDNA from the total RNA was used as template for the PCR reaction. A negative control was run without adding the reverse transcriptase enzyme, and a positive control was run on genomic DNA (gDNA). PCR reactions were performed using the AccuPrime™ Taq DNA Polymerase High Fidelity kit (Invitrogen), adapted to amplify DNA fragments up to 20 kbp. The PCR conditions were as follows: 30 sec of initial denaturing at 94°C, 36 amplification-denaturing cycles lasting 30 sec at 94°C, annealing for 30 sec at 47°C or 43.5°C, depending on the melting temperature of the primers, and extension at 68°C, for 2 to 5 min, depending on the amplicon length. The total reaction volume was 50 μl, and 10 μl of this mix were analyzed on 1% agarose gel stained with Ethidium Bromide. 17 pairs of genes were also tested with 26 amplification-denaturing cycles.

## Results

### *Buchnera *operon map

**Figure 3:**
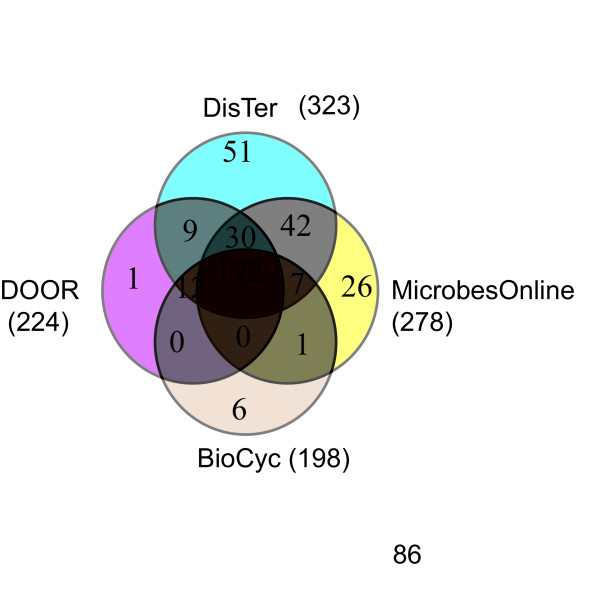
**Comparison of the four predicted transcription unit collections of *Buchnera***. Four-way Venn diagram of the pairs classified as STUs (Same Transcription Unit, see Methods) by the different methods. No *Buchnera *gene pair was classified as STU by the MicrobesOnline and DOOR methods but as DTU (Different Transcription Units, see Methods) by the other two. Nor was any *Buchnera *gene pair classified as STU by the DisTer and BioCyc methods and as DTU by the other two. The number shown below the method name indicates the total number of *Buchnera *gene pairs classified as STU by the method.

The entire *B. aphidicola *genome is arranged into 443 adjacent genes pairs having the same strand orientation, and 167 pairs of adjacent genes with the opposite strand orientation. Our prediction (DisTer) was run on the 443 adjacent gene-pairs, and the results were compared to the predictions available in the literature for *B. aphidicola *(Figure 3). The DisTer prediction differed significantly from each of the other predictions. MicrobesOnline was closest to the DisTer prediction, but even these two methods disagreed on the classification of 99 out of the 443 same-strand pairs (Figure 3).

We therefore propose a new operon map of the *B. aphidicola *genome, containing the 133 predicted polycistronic TUs out of the 288 found in the *B. aphidicola *genome (155 are monocistronic). The list of the 288 TUs of *B. aphidicola *is presented in the Additional File 4. The average length (number of genes) of the predicted *B. aphidicola *TUs is 2.12 (1.63 in *E. coli*), whereas the average length of its polycistronic TUs is of 3.43 genes (3.17 in *E. coli*). On average, *B. aphidicola *TUs contain more genes than those of *E. coli*, and the TU length distribution of *B. aphidicola *is significantly shifted to the right when compared to that in *E. coli *(Wilcoxon test, p-value ≈ 10^-9^, Figure 1).

### Experimental validation of the *Buchnera *operon map using microarray data

In order to compare the four predicted collections of TUs of *B. aphidicola *(DisTer, MicrobesOnline, DOOR and BioCyc), we used gene expression data obtained by Reymond et al. [12], and we made the assumption that the variability of gene expression for the STU (Same Transcription Unit, see the Methods section) pairs would be lower than that of the DTU (Different Transcription Unit) pairs. The comparison was carried out after excluding monocistronic TUs, and using one-way ANOVA-model with the polycistronic TUs as the qualitative regression variable and the log-transformed expressions as the explained variable (Table 1).

**Table 1:** One-way ANOVA analysis of gene expression data for comparing the operon maps of Buchnera.

	DisTer	BioCyc	MicrobesOnline	DOOR
Number of predicted STU pairs	323	198	278	224
Total number of predicted TU	288	413	333	387
Adjusted R^2^	0.44	0.38	0.40	0.41
Non-parametric p-value	<1e-04	0.0143	1e-04	0.0026

As the four predicted operon maps have different numbers of polycistronic units, the adjusted R^2 ^values (penalized by the number of parameters) were compared. Although the four methods gave similar adjusted R^2 ^values, DisTer showed slightly greater correlation (Table 1). P-values were computed using a non-parametric approach: we simulated 10,000 other *B. aphidicola *operon maps by shuffling the TU labels in such a way that the same TU lengths were conserved as in the original map. For each simulation, the one-way ANOVA F-value was calculated. The non-parametric p-value is the proportion of the simulated F-values that are higher than the observed F. Although all four methods gave significant p-values, the lowest value was obtained with DisTer (Table 1).

### Experimental validation of *Buchnera *operons using RT-PCR

**Figure 4:**
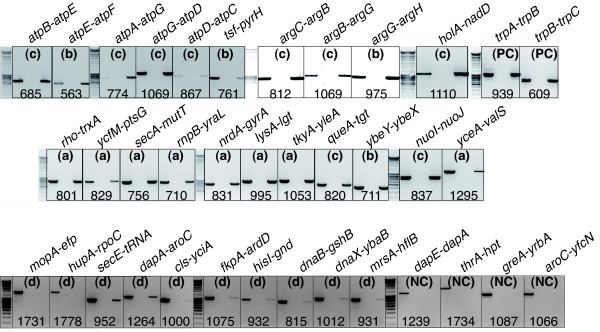
**Experimental testing of *B. aphidicola *gene pair status (STU or DTUs), by RT-PCR amplification using specific primers (third lane)**. For each gene pair amplification we used a positive control, in which gDNA was used instead of cDNA for the PCR reaction (first lane) and a negative control, for which the reverse transcriptase was omitted from the RT reaction (second lane). The size (bp) of the amplicon is shown below each gene pair. (a) gene pairs predicted as STU solely by DisTer; (b) gene pairs predicted as STU by DisTer with no consensual annotation given by the 3 other methods; (c) gene pairs predicted as STU by all methods; (d) gene pairs predicted as DTUs by DisTer; (PC) positive controls; (NC) negative controls.

To confirm the co-transcription of the genes predicted within STU pairs (*i.e.*, the presence of the polycistronic mRNA), RT-PCR reactions were performed for some gene pairs using primers that amplify across their intergenic region. Each amplicon contained the intergenic region of the tested pair, and at least 300 bp of the flanking regions located upstream and downstream of this intergenic region. In order to verify if our technique allows distinguishing between STU and DTU gene pairs, we used two positive controls (Figure 4, PC-pairs) and 4 negative controls (Figure 4, NC-pairs). The positive controls were chosen among the gene pairs of the operon *trpABCD*, which had previously validated by Bauman et al. [30]. The negative controls were chosen among the convergent and divergent pairs of *B. aphidicola *(3 divergent and one convergent pair). The products corresponding to the positive control pairs were amplified by RT-PCR, but not the products corresponding to the negative control pairs. Thus this technique is appropriate for experimentally verifying the DisTer predictions. We tested the predictions made for 31 gene pairs: 8 pairs predicted as STU solely by DisTer (Figure 4, a-pairs), 4 pairs predicted as STU by DisTer and only one or two of the other methods (Figure 4, b-pairs), 9 pairs that were predicted as STU by all the 4 methods (Figure 4, c-pairs) and finally, 10 pairs of adjacent genes predicted as DTUs pairs by DisTer (Figure 4, d-pairs). Twenty-nine out of the 31 experimentally tested pairs revealed a corresponding mRNA (Figure 4 and Additional File 5). Eight pairs out of the 10 pairs predicted as DTUs pairs by DisTer, but also by the other methods, (except the *dnaX*-*ybaB *pair) were amplified by RT-PCR. This experimental result suggests that even if DisTer predicts longer operon structures in *Buchnera *as compared to the 3 other predictors, the real *Buchnera *operon map may by even more polycistronic.

We also detected the mRNA for the four following long polycistronic TUs: *atpBEFHAGDC, argCBGH*, *trpABCD *and *leuSholAnadDsirA *(Additional Files 6), although we were not able to amplify the complete mRNA from the *atp *operon (the longest), but only all the contiguous overlapping fragments.

### *B. aphidicola *vs. *E. coli *TU comparison

Three studies have established the gene repertoire of the last common ancestor of *E. coli *and *B. aphidicola *[10,31,32]. However, so far we do not know enough to reconstruct the ancestral TUs. In this situation, we used the *E. coli *TUs as a benchmark in our study. *E. coli *orthologues were identified for more than 95% of the genes of *B. aphidicola *(with the exception of 3 orphan genes, and 15 tRNAs with ambiguous orthologues), which facilitated comparison of the two bacteria. Using *B. aphidicola *TUs as the comparison start point for the *B. aphidicola*/*E. coli *comparison, we defined five TU types (identical, similar, split, merged and reorganized). The compositions of these TUs are given in Table 2, and schematized in the Additional File 7.

**Table 2:** Characterization of the TUs of Buchnera predicted by DisTer.

TU type	Number of TUs	Number of genes	Monocistronic TUs	Polycistronic TUs
Identical	121	162	99	22
Similar	54	88	38	16
Split	23	47	11	12
Merged	64	231	0	64
Reorganized	16	70	0	16

**Identical TUs **are *B. aphidicola *TUs with exact orthologous replicates in *E. coli*. These identical TUs have not been internally modified by genomic rearrangements or sequence evolution during the evolution of these two lineages. There are 121 identical TUs, 99 of which are monocistronic. The identical monocistronic TUs are evenly distributed on the *B. aphidicola *chromosome, and 78% of them do not belong to a syntenic fragment (a syntenic fragment designates a set of adjacent genes that have the same organization, order and strand orientation in the two genomes); the remaining 22% are located within syntenic fragments, and 10 of them form 5 pairs of adjacent syntenic TUs. The polycistronic identical TUs contain mainly genes coding for enzymes (or enzyme subunits), ribosomal proteins or ATP/GTP binding proteins.

**Similar TUs **are *B. aphidicola *TUs for which the orthologous TUs replicates in *E. coli *are longer because they include genes which have no orthologues in *B. aphidicola*. Most of the similar TUs, like the identical ones, are monocistronic (Table 2). Their orthologous TUs in *E. coli *contain another 80 genes not found in the *B. aphidicola *genome. In order to find out whether an *E. coli *gene was present in the ancestor genome and had been lost in *B. aphidicola *or acquired in the *E. coli *lineage, we checked for the presence of this gene in the common ancestor genome proposed by Silva et al. [32], or alternatively, in *V. cholerae*, *H. influenzae *or *P. aeruginosa*. If it was found in any of these, we assumed that the gene had been present in the ancestor genome, and had been lost in the *Buchnera *lineage. Hence, 63 of these 80 genes (79%) were found to have been lost in the *B. aphidicola *lineage.

Thirty-eight similar TUs are monocistronic, 30 (80%) of them do not belong to any syntenic fragment, which means that, in addition to deletions in the *B. aphidicola *lineage, there had also been genomic rearrangements, which had changed the genomic context of the conserved genes, possibly in both lineages. Among the 16 polycistronic similar TUs, 6 have lost the first gene of the TU (and so their ancestral promoter and regulation), 3 have lost their final gene(s), and 5 their internal gene(s). The 2 remaining TUs have no adjacent losses. The main mechanism by which similar TUs have appeared in *B. aphidicola *is gene deletion, but local sequence evolution is also important, *e.g.*, there are polycistronic similar TUs corresponding to ancestral TUs that have lost their middle gene, which implies that selection pressure exerted on regulatory elements has preserved the operon structure following elimination of the gene.

**Split TUs **are *B. aphidicola *TUs for which the orthologous TU replicates in *E. coli *are longer, including genes with orthologues from other TUs in *B. aphidicola*. It is striking that the *B. aphidicola *split TUs corresponding to fragments of the same *E. coli *TU remain adjacent on the *B. aphidicola *chromosome. Seventeen of the 23 genes belonging to these TUs in *E. coli *that are not found in *B. aphidicola *have been lost in *B. aphidicola *lineage; among the other 6 genes, 2 (*ygdK *and *pheM*) have been acquired in the *E. coli *lineage. The 4 remaining genes code for structural RNA, and their orthology cannot be established unambiguously. Hence, the split TU are clearly examples of TUs that have evolved exclusively through local sequence evolution in one or both lineages.

**Merged TUs **are *B. aphidicola *TUs for which orthologous genes in *E. coli *belong to several TUs, sometimes accompanied by other genes with no orthologues in *B. aphidicola*. By definition, there are no monocistronic TUs in this class. Among the 64 merged TUs of *B. aphidicola*, only 18 correspond to adjacent TUs in *E. coli *(as a result of border reorganization). Ninety-nine genes belonging to *E. coli *merged TUs were not found in the *B. aphidicola *genome, and 71 of these genes have also been lost in the *B. aphidicola *lineage. These merged TUs are examples of TUs that have primarily been shaped by genome rearrangements rather than by local sequence evolution. The *E. coli *orthologues of 33 of the 64 merged TUs of *B. aphidicola *are not specifically regulated in *E. coli *(*i.e*. constitutively transcribed in *E. coli*).

**Reorganized TUs **are *B. aphidicola *TUs for which the orthologous genes in *E. coli *belong to different TUs. The difference between these reorganized TUs and merged TUs is that the *E. coli *genes for which orthologues are not found in the orthologous reorganized TUs in *B. aphidicola*, are found in another TU in the *B. aphidicola *genome. Among the 30 genes belonging to orthologous *E. coli *reorganized TUs and not found in *B. aphidicola*, 27 have been lost from the *B. aphidicola *lineage. Six of *B. aphidicola *reorganized TUs are composed of ancestral gene pairs (the ancestral pairs were identified on the basis of their OperonDB score; ancestral pairs have scores of above 86%), and so have probably been reorganized in the *E. coli *lineage. The *E. coli *orthologues of 7 of the 16 reorganized TUs are not regulated by specific transcription factors in *E. coli*.

### *Buchnera *operon map evolution - Local and general dynamics

**Figure 5:**
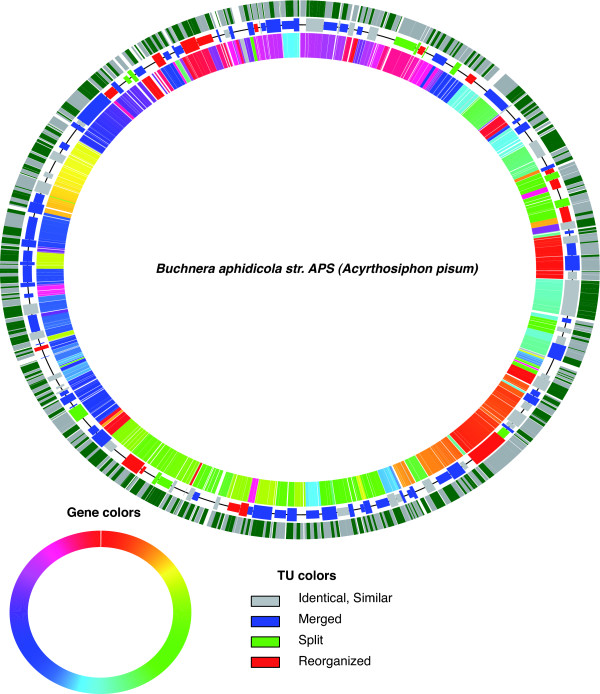
**Operon and genomic maps of *Buchnera***. The small circle portrays a simplified *E. coli *map (only genes having an orthologue in the *Buchnera *genome were used in drawing the map), each gene is specifically colored (consecutive genes have proximal colors). The inner circle depicts the *Buchnera *genome; the same colors are used for the genes as for their orthologues in *E. coli*. The middle circle is the operon map of *Buchnera*, in which only the polycistronic TUs are shown. The outer circle shows the consecutive blocks of adjacent genes in *Buchnera *of which the orthologues in *E. coli *are also adjacent and belong to a single TU. The blocks are colored alternately in grey and green in order to make it possible to distinguish between two adjacent blocks. The synteny map of *Buchnera *(versus *E. coli*) is not shown here; nevertheless the gene color gives some insight into the multiple genomic rearrangements that have occurred since the lineages diverged.

Among the 611 pairs of adjacent *B. aphidicola *genes, 320 pairs (formed by 441 genes) are ancestral pairs: these are gene pairs that are either present in *B. aphidicola *and in *E. coli *(237), or in *B. aphidicola *and in more distant bacteria (83). These ancestral pairs are scattered around the chromosome, and tend to conglomerate on 68 fragments containing an average of 6.5 genes in *B. aphidicola*. Hence, the *B. aphidicola *genomic map is an alternation of ancestral fragments and reorganized fragments (2.5 genes long in average). Operon and genomic maps of *Buchnera *are shown in Figure 5.

Among the 237 pairs present in both *E. coli *and *B. aphidicola*, 188 (79.3%) are STU pairs; 15 (6.3%) are DTU pairs, and 34 (14.4%) have opposite status in the two lineages (Additional File 8). Hence, most of the ancestral gene pairs conserved in both bacterial lineages are STU pairs. The 188 ancestral gene-pairs are included in TUs belonging to the various TU classes defined above: 41 (21.8%) in identical TUs, 27 (14.4%) in similar TUs, 22 (11.7%) in split TUs, 68 (36.2%) in merged TUs and 30 (15.9%) in reorganized TUs.

The genes of the remaining 83 ancestral pairs (not adjacent in *E. coli*) are part of TUs that result from genomic rearrangements in the *E. coli *lineage, including 18 pairs belonging to TUs in *E. coli *which are clear examples of gene insertion, since the genes of these 18 pairs belong to a same TU in the bacterium.

### Characteristics of *Buchnera *TUs

#### Intergenic distances

**Figure 6:**
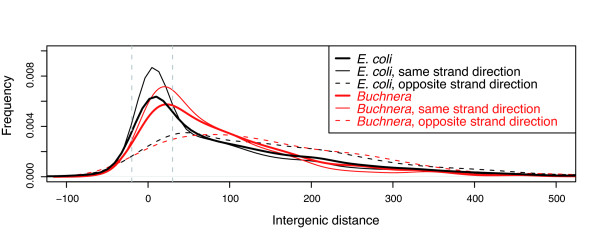
**Intergenic distance distributions in *Buchnera *and *E. coli***. The two vertical lines identify the -20 bp and +30 bp regions. The same class of intergenic distance distributions (global, same strand direction and opposite strand direction) were compared between *Buchnera *and *E. coli*.

Intergenic distance distribution in bacterial genomes possessing polycistronic TUs generally shows a characteristic peak between -20 and +30 bp, suggesting that operons are universally compact (apart from some cases of complex operons using alternative promoters) [33]. *B. aphidicola *is not an exception to this rule (Figure 6). Nevertheless, the intergenic distance distribution of *B. aphidicola *is different from that of *E. coli *(Wilcoxon test, p-value = 0.05). Since the opposite strand distributions are similar (Wilcoxon test, p-value = 0.78), this difference results from the comparison between the same strand distributions (Wilcox test, p-value = 8*10^-4^): a slight shift to the right is observed for *B. aphidicola *(Figure 6). More precisely, *B. aphidicola *presents fewer gene overlaps (negative distances), and more intergenic distances ranging from 20 to 100 bp than *E. coli*. Moreover, *B. aphidicola *has fewer intergenic distances of 220-300 bp and, hence, shorter average intergenic distances (Chi2 test with Holm corrected p-values).

**Figure 7:**
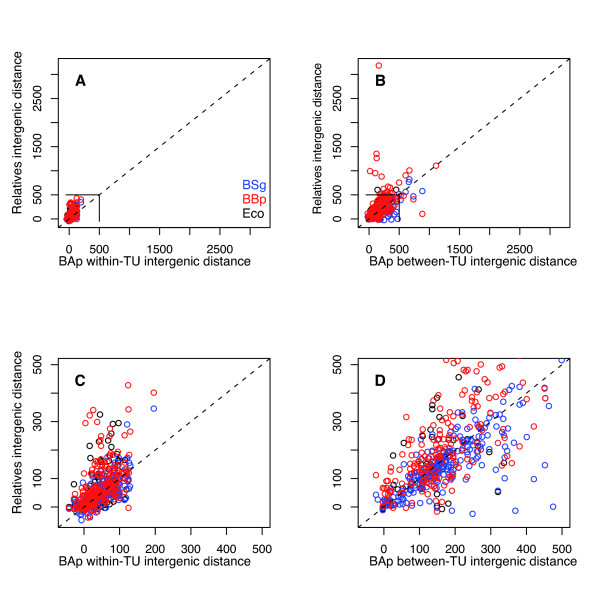
**Comparison of orthologous intergenic distances between BAp, BSg, BBp and *E. coli***. Orthologous intergenic distances in *E. coli*, BSg and BBp are represented as a function of intergenic distance in BAp. A, C: BAp within-TU intergenic distance, B and D: between-TU intergenic distance. C and D zoom-in versions of the top graphics (A, B) in the 0 bp - 500 bp region.

Among the 521 overlapping gene pairs in *E. coli*, only 28 (5%) are found in *B. aphidicola*, and 20 of them also overlap in *B. aphidicola*. On the other hand, *B. aphidicola *has a significantly smaller intrinsic proportion of overlapping adjacent gene pairs (6%) than *E. coli *(13%), highlighting the fact that the process of overlapping coding sequences has rarely occurred during the evolution of the *B. aphidicola *lineage. More generally, we compared the "orthologous" intergenic distances between *E. coli*, and *B. aphidicola *from *Schizaphis graminum *(BSg), *B. aphidicola *from *Baizongia pistaciae *(BBp) and *Acyrthosiphon pisum *(BAp). I defined an "orthologous" intergenic region as a region between two adjacent genes in *B. aphidicola *that share adjacent orthologues in the other strains of species (Figure 7). The higher variability of the between-TU distances as compared to that of the within-TU distances for the 3 *B. aphidicola *strains and *E. coli *indicates that some additional constraints must be controlling the evolution of the within-TU distance (Figure 7C and 7D).

**Figure 8:**
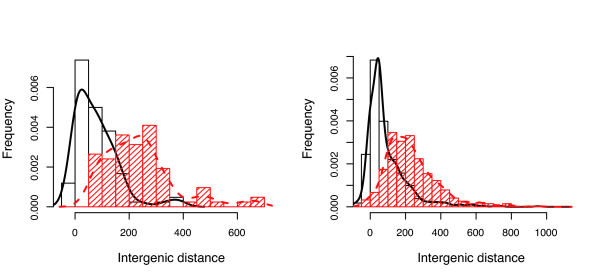
**Comparison of the convergent and divergent intergenic distance distributions**. Comparison of the convergent intergenic distance distribution (black) and divergent intergenic distance distribution (red) for *Buchnera *(left) and *E. coli *(right).

As we have already mentioned, the intergenic distance of opposite strand pairs are similar in *B. aphidicola *and *E. coli*, even when convergent and divergent pairs are considered separately. The pronounced differences existing between the convergent and divergent pair distances (the divergent pair intergenic distances being longer than the convergent ones) were found in both organisms (Figure 8 and Additional File 9).

#### Promoters

Small-genome bacteria seem to contain a smaller proportion of regulatory elements than large-genome ones [34,35]. However, we searched the consensus sequence of the constitutive σ^70 ^promoter in the 500 bp upstream of the coding sequence of each *Buchnera *gene, using Bprom software, mentioned above. Contrarily to the previous hypothesis of promoter losses in *Buchnera *[31], we found significant joint -10 and -35 σ^70 ^motives upstream of 98% of the *Buchnera *TUs. We also found putative alternative promoters σ^70 ^promoters (*i.e.*, promoters associated to inner genes of operons), but with a significantly lower score of prediction (Additional File 10).

The significant difference between the convergent and divergent intergenic distances we detected, also supports the hypothesis that some specific constrained areas (that might bind transcription regulators) are maintained in the upstream region of some genes.

#### Terminators

**Figure 9:**
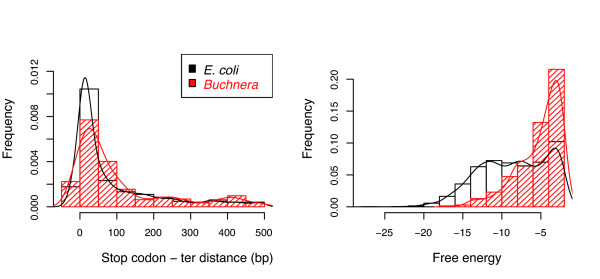
**The *Buchnera *and *E. coli *stop codon-terminator distance distributions (left) and the hairpin free energy distributions (right)**.

The predicted terminators are located downstream of the coding sequences. The distribution of the distance between the stop codon and the predicted terminators shows a peak at 13 bp for *E. coli *and at 26 bp for *B. aphidicola*, with a general shift to the right of the distance distribution in *B. aphidicola *(Figure 9, Wilcoxon test, p value ≈ 10^-5^). *B. aphidicola *terminators are also less stable (i.e. the absolute value of the free energy of their hairpins is lower) than *E. coli *(Figure 9, Wilcoxon test, p value ≈ 10^-16^). We also analyzed the type of intergenic regions in which the terminators have been predicted (Table 3). There is a visible trend for the terminators to be present in convergent intergenic regions rather than in the same strand intergenic regions. Indeed, the convergent intergenic regions correspond to the end of two TUs, and are therefore likely to contain at least one terminator. Sometimes a single terminator (bidirectional) is sufficient for the transcription termination of two adjacent genes [36], and of course, the terminators are not really necessary in the divergent intergenic regions. Overall, *B. aphidicola *and *E. coli *have the same proportion of predicted terminators within the different type of intergenic region (Table 3, same strand intergenic regions: Chi2 test, p-value = 0.143, convergent intergenic regions: Chi2 test, p-value = 0.406).

**Table 3:** Number of terminators predicted in the different types of intergenic regions in Buchnera and E. coli.

Intergenic region type	*Buchnera*	*E. coli*
Same strand, total	443	3194
Same strand, predicted ter (%)	209 (47)	1385 (43)
Convergent, total	84	688
Convergent, predicted ter (%)	72 (86)	615 (89)
Divergent, total	83	688
Divergent, predicted ter (%)	0 (0)	0 (0)

### Operon structure and the evolution of the coding sequence length

The relation between the operon structure and the dynamics of the coding sequence length was analyzed within the *E. coli *and *B. aphidicola *lineages, using external outgroups to identify the direction of any evolutionary changes (see Methods section). Hence, there are 122 genes out of 597 in *B. aphidicola *that have the same length in *E. coli*. For the 412 remaining *B. aphidicola *genes, the direction of change in length evolution has been identified: 57 (66) were found to have increased (or decreased) in the *E. coli *lineage, and 96 (193) were found to have increased (decreased) in the *B. aphidicola *lineage. Hence, more of the *B. aphidicola *coding sequences have shortened during their evolution in the *B. aphidicola *lineage as compared to those in the *E. coli *lineage, as previously reported by Charles et al. [37].

**Figure 10:**
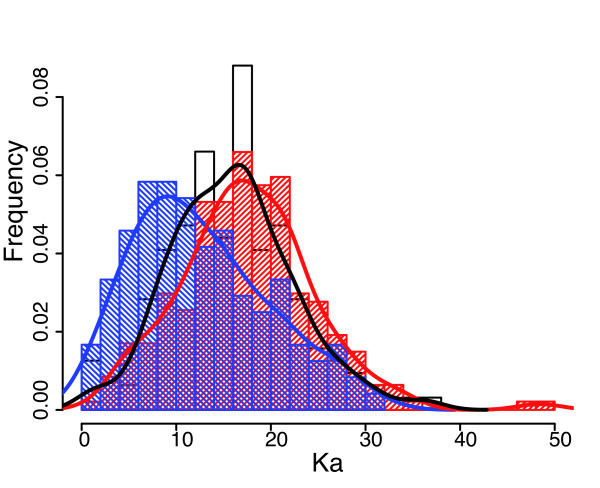
**Comparison of the distributions of Kas as a function of the evolution type of coding sequence length**. Three Ka distributions are represented: Ka distribution of *Buchnera *genes that have the same length as their *E. coli *orthologues (blue), that are shorter than their *E. coli *orthologues (red) and are longer than their *E. coli *orthologues (black curve). The distributions shown in black and red are significantly different from the one shown in blue.

A marked difference in evolutionary constraints was observed between genes with longer or shorter sizes in *B. aphidicola *than in *E. coli *(Figure 10, Kruskal-Wallis test, p-value ≈ 10^-9^), *i.e.*, genes of which the sequence size is evolving display a corresponding change in their composition. The *B. aphidicola *genes that are shorter than their *E. coli *orthologues belong to one of the two following classes: either the coding sequence has shrunk in the *B. aphidicola *lineage, or the coding sequence has lengthened in the *E. coli *lineage. Based on the calculation of Ka, it was not possible to detect any difference between these two populations of genes in the selection constraints. The same was observed for *B. aphidicola *genes that are longer than their *E. coli *orthologues (Additional File 11). Finally, no TUs effect was observed on the evolution of the length of the coding sequences.

## Discussion

We have predicted a new operon map for BAp, which contains 133 polycistronic TUs. The novelty of our prediction is that we used the presence of the transcription terminators. More generally our prediction relies entirely on structural criteria (using only the intergenic distance and terminator presence), whereas the existing predictions use functional similarity, metabolic activity correlation and/or orthology information in addition to the intergenic distance criterion. The functional clustering of genes on chromosome is one of the hypotheses used to explain the origin of operons. Accordingly, using the functional similarity of adjacent genes is a natural choice for TU prediction. However, this approach might miss TUs created by genome reorganization and which have led to more recent TUs composed of functionally unrelated genes (*i.e*., TUs from the merged class and possibly some of the reorganized class). Indeed, our method did detect some specific *B. aphidicola *polycistronic TUs, probably generated by the genomic rearrangements occurring during the early stages of symbiosis establishment that are known to be characterized by high genome dynamics, and assembling genes with no functional similarities. It was reported that this kind of polycistronic TUs are rapidly lost in free-living bacteria [38]. In *B. aphidicola*, on the contrary, it seems that such functionally unrelated TUs were further fixed as a result of the loss of the recombination machinery during the process of genome shrinkage [39,40]. We also experimentally validated several of these polycistronic TUs that were predicted exclusively by DisTer. This supports our decision to use only structural features for predicting operons.

The *B. aphidicola *operon map predicted by DisTer is more compact that the other 3 available in the literature; it is also more compact than that of *E. coli *(Table 1). On average, each *B. aphidicola *TU contains 2.12 genes, whereas *E. coli *contains fewer genes per TU (1.63), *E. coli *having a higher proportion of monocistronic TUs. Also, even though the DisTer predicted map is the more compact, the real map may be even more compact (as suggested by our predicted DTU pairs), due to the originality of the *B. aphidicola *genome.

The predicted TUs correlate well with the predicted σ^70 ^promoters (Additional File 10) as well as with expression data (*i.e.*, the expression of genes within a given TU is more similar than that of genes belonging to two different TUs). Part of the gene expression correlation is probably explained solely by gene proximity; however, the construction of our null hypothesis (Table 1) by shuffling TU-labels while preserving gene proximity reveals that the TU borders (*i.e.*, gene promoters and terminators) significantly partitioned gene expression in *B. aphidicola*. Moreover, for 15 TUs for which there were contradictory predictions in the literature, we have experimentally validated the presence of a polycistronic mRNA in *B. aphidicola*.

The experimental technique we used to verify the DisTer predictions was validated by using positive and negative controls. The former were amplified by RT-PCR while the later were not. All the gene pairs predicted as STU by DisTer (but not necessarily by the other three methods) were amplified by RT-PCR. Surprisingly, only 2 out of the 10 predicted DTUs pairs were experimentally confirmed as such, suggesting an even more compact operon map. The lack of the prediction power concerning the other 8 pairs can be explained by the fact that DisTer, and the 3 other predictors are trained on *E. coli *genome and/or used common traits of known bacterial operons that might not reflect the overall characteristics of the *Buchnera *genome (AT bias, loss of recombination and intracellular living).

Since the *B. aphidicola *genome is an *E. coli *subset, we were able to compare the TUs of these two organisms and shed light on the dynamics of TUs in both lineages, thus revealing insights about operon map evolution. Operon map changes must certainly have occurred as a result of global reorganization (recombination, inversion, translocation) at the level of the reorganized fragments, but also inside the ancestral fragments (gene deletions) or even at the borders between fragments (border reorganizations) as a result of local sequence evolution. There are two types of operon map changes: modifications of the TU content, and the reordering of the TUs on the map. The former type will necessarily have an impact on gene regulation, as it will always result in a change the co-transcribed gene sets, whereas the second one may have a more fuzzy influence on gene transcription (*e.g.*, the accessibility of the transcription machinery to the TU may change), without necessarily altering gene regulation (*e.g.*, conserving the same promoter).

The *B. aphidicola *operon map seems to have been reorganized initially by genome rearrangements: more than 45% of the TUs were affected by these processes. We also found that some changes had occurred in the *E. coli *lineage, confirming that genome rearrangement is one of the most important processes in operon map evolution.

The second way the operon map has evolved is related to local sequence evolution, and more precisely to intergenic sequence evolution. Intergenic regions are known to be evolving more rapidly than coding sequences. These regions contain structural elements, such as promoters and terminators. Thus, the intergenic sequence evolution acts at a very local level, and causes inter-TU border fluctuations (*e.g.*, the split TUs we mentioned above).

These two generic evolutionary processes do not act either independently, or sequentially, and the operon maps we are studying result from their joint influence. As a result, it is generally impossible to quantify the contribution made by each process in the reorganization of the operon map, except to say that no further rearrangements events are occurring in the *B. aphidicola *genome, since it has lost the necessary elements. This means that the operon map of *B. aphidicola *is solely governed by local sequence evolution since its genome has entered genomic stasis [40].

Moran et al. [41] showed recently that *B. aphidicola *sequence evolution is not symmetrical, and its sequences can only evolve towards shrinkage, since the observed insertions are not bigger than a few bp, and are mainly caused by polymerase slippage. However, it does seem as if *B. aphidicola *can evolve only towards a more polycistronic operon map, since disintegration of the promoter and/or terminator will induce the formation of new polycistronic TUs or the extension of existing TUs, while the remaining polycistronic TUs remain unaltered, unless one of their genes is gradually deleted by a pseudogenization process. The number of merged TUs and the number of genes they are assembling (231, more than 30% of *B. aphidicola *genome) are consistent with this hypothesis.

The *B. aphidicola *intergenic distance analysis (within and between TUs) also sheds lights on some other aspects of the *B. aphidicola *operon map. One finding is that *B. aphidicola *coding sequences show less superimposition than *E. coli *coding sequences. The superimposed coding sequences are generally found in ancient TUs favoring the coupling of the translation for the genes within a same operon [38]. Hence, the recent reorganization of the *B. aphidicola *operon map could explain the lack of superimposition in the bacterial genome. Moreover, no specific superimpositions appeared in *B. aphidicola*. One explanation is that *B. aphidicola *might not be able to create new superimposed sequences whether because of the strong AT bias preventing dual coding of both the 5' and the 3' end on different reading frames, whether because of the loss of selective constraints on its genome compaction.

The *B. aphidicola *positive intergenic distances are shorter on average than those of *E. coli*, although the intergenic distances between adjacent genes with opposite strand directions are similar in *E. coli *and *B. aphidicola*. It is striking however to note that, as in *E. coli*, convergent intergenic distances are shorter than divergent ones in *B. aphidicola *(Figure 8). An hypothetical explanation for this phenomenon is that the divergent intergenic regions constitute the physical support of two promoters, and that they therefore need more space than a convergent region, which is supposed to contain only terminators [42]. The hypothesis of the degenerated or inexistent promoters was proposed for the *B. aphidicola *model, but there are still some selection constraints on the intergenic sequences length, which suggest that there must be some structural genomic element that is important for *B. aphidicola *gene transcription.

Finally, we investigated the impact of gene regulation on operon map dynamics. For this purpose, we first studied the *E. coli *TUs containing at least one gene for which an orthologue was found in the *B. aphidicola *genome. Our results suggest that the assembly of the *B. aphidicola *genes in TUs had not been constrained by their ancestral specific regulation (as far as this can be accessed from the specific regulation of their orthologues in *E. coli*). Indeed, similar TU distributions among the 5 classes defined above were observed for *B. aphidicola *TUs including genes with orthologues which are either regulated or un-regulated in *E. coli*, (Additional File 12). Two hypotheses could explain this observation: either most *E. coli *regulation mechanisms were established after the lineage divergence, or some *E. coli *specific regulations are ancestral in origin, but they did not correspond to the demand for *B. aphidicola *regulation, and so the operon map dynamics of this latter species was not influenced by specific ancestral regulations. On the other hand, a correlation was observed between gene regulation in *E. coli *and gene conservation in *B. aphidicola*, which was significantly greater (Additional File 13, Chi-2 test, p-value = 0.008) if the genes belonged to a regulated TU in *E. coli *(23%), than to a non-regulated one (18%).

## Conclusions

*B. aphidicola *seems to be evolving towards a map enriched in polycistronic TUs. Some of these polycistronic TUs seem to be accidental, without any strong evolutionary value, and therefore passively maintained. This observation is very important for understanding the selection pressure exerted on *B. aphidicola *gene expression. The fact that co-expression is maintained, even when it is unnecessary, suggests that in *B. aphidicola *what is important is that gene expression should occur (in a binary fashion) and not that the specifically tuned regulation should occur. The fact that the need for specific regulation of TUs exerts some pressure on gene conservation and gene colocalization, but not on gene assembly in the operon map, also supports this hypothesis of evolution of the operon map by genetic drift. However, the work of Tamames *et al*. [43] on the modularity of the protein interaction network of *B. aphidicola *suggests selection constraints on its operon map evolution. Indeed, the interactome in *B. aphidicola *is highly diminished and proteins present very similar interaction numbers. As a consequence of the need for similar abundances, protein-coding genes should be expressed at similar levels, which is possible by ensuring their co-expression within operon structures. Functional analyses, including proteomics and interactome reconstruction, would be needed to explore further the forces driving the evolution of *B. aphidicola *operon map and its gene-regulation network.

## Authors' contributions

LB carried out a part of the experiments, developed the transcription unit predictor, performed the analysis and drafted the manuscript. FC participated in the design of the experimental study, and helped to draft manuscript. GD and KG carried out a part of the experiments. CG participated in statistical analysis and interpretation of results. HC conceived the study, participated in the statistical analysis and helped to draft manuscript. All authors read and approved the final manuscript.

## Supplementary Material

Additional file 1 **Definition of the adjacent gene-pairs types**. Click here for file

Additional file 2 **TU predictor models evaluated during DisTer construction**. Click here for file

Additional file 3 **Quality of the predictions for the three predictor models of DisTer**. Click here for file

Additional file 4 **List of the *B. aphidicola *TUs predicted with DisTer**. Click here for file

Additional file 5 **Pairs of adjacent genes for which the STU status was experimentally validated by RT-PCR**. Click here for file

Additional file 6 **Experimental validation by RT-PCR for 4 complete operons**. Click here for file

Additional file 7 **Description of the *Buchnera *TU types defined by comparison with homologous *E. coli *TUs**. Click here for file

Additional file 8 ***Buchnera *vs. *E. coli *gene pair status comparison**. Click here for file

Additional file 9 **Intergenic distance characteristics for each pair type**. Click here for file

Additional file 10 **Distributions of the scores of the predicted σ^70 ^promoters**. Click here for file

Additional file 11 ***Buchnera *gene Ka distributions**. Click here for file

Additional file 12 **Distribution of *E. coli *TUs in the *Buchnera *TU classes**. Click here for file

Additional file 13 **Counts of ancestral genes conserved or not conserved in *Buchnera *depending on their regulation in *E. coli***. Click here for file
