# Supplementary material for: Structure and dynamics of the operon map of Buchnera aphidicola sp. strain APS
Source: BMC Genomics. 2010 Nov 25;11:666. doi: 10.1186/1471-2164-11-666 (PMC3091783; doi:10.1186/1471-2164-11-666)
Supplement: Additional file 1 — Definition of the adjacent gene-pairs types. [file 1471-2164-11-666-S1.PDF]

Definition of the adjacent gene-pairs types

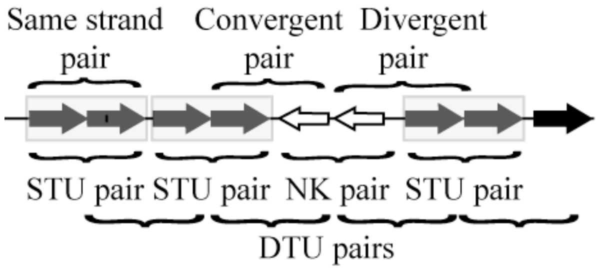

Definition of the three types of adjacent gene pairs: same strand, convergent and divergent pairs regarding gene strands indicated by the arrow direction; definition of same TU pair (STU) and different TU pair (DTU) and pairs with unknown operon status (NK). The rectangles surrounding the genes indicate the experimentally determined TU.

Structure and dynamics of the operon map of *Buchnera aphidicola* sp. strain APS

Lilia Brinza, Federica Calevro, Gabrielle Duport, Karen Gaget, Christian Gautier, Hubert Charles
