# Supplementary material for: Structure and dynamics of the operon map of Buchnera aphidicola sp. strain APS
Source: BMC Genomics. 2010 Nov 25;11:666. doi: 10.1186/1471-2164-11-666 (PMC3091783; doi:10.1186/1471-2164-11-666)
Supplement: Additional file 2 — TU predictor models evaluated during DisTer construction. [file 1471-2164-11-666-S2.PDF]

TU predictor models evaluated during DisTer construction

1. Intergenic distance and the presence of the terminator, assuming that these properties are independent;

$$p(\mathrm{STU} \mid D = d, T = t) = \frac{p(\mathrm{STU}, D = d, T = t)}{p(D = d, T = t)}$$
$$= \frac{p(D = d, T = t \mid \mathrm{STU})\, p(\mathrm{STU})}{p(D = d, T = t \mid \mathrm{STU})\, p(\mathrm{STU}) + p(D = d, T = t \mid \mathrm{DTU})\, p(\mathrm{DTU})}$$
$$= \frac{p(D = d \mid \mathrm{STU})\, p(T = t \mid \mathrm{STU})\, p(\mathrm{STU})}{p(D = d \mid \mathrm{STU})\, p(T = t \mid \mathrm{STU})\, p(\mathrm{STU}) + p(D = d \mid \mathrm{DTU})\, p(T = t \mid \mathrm{DTU})\, p(\mathrm{DTU})}$$

2. Joint distribution of the intergenic distance and the presence of the terminator;

$$p(\mathrm{STU} \mid D = d, T = t) = \frac{p(D = d, T = t \mid \mathrm{STU})\, p(\mathrm{STU})}{p(D = d, T = t \mid \mathrm{STU})\, p(\mathrm{STU}) + p(D = d, T = t \mid \mathrm{DTU})\, p(\mathrm{DTU})}$$

3. Joint distribution of the intergenic distance and the TransTermHP score of the terminator;

$$p(\mathrm{STU} \mid D = d, Tscore = s) = \frac{p(D = d, Tscore = s \mid \mathrm{STU})\, p(\mathrm{STU})}{p(D = d, Tscore = s \mid \mathrm{STU})\, p(\mathrm{STU}) + p(D = d, Tscore = s \mid \mathrm{DTU})\, p(\mathrm{DTU})}$$

Structure and dynamics of the operon map of *Buchnera aphidicola* sp. strain APS

Lilia Brinza, Federica Calevro, Gabrielle Duport, Karen Gaget, Christian Gautier, Hubert Charles
