# Supplementary material for: Structure and dynamics of the operon map of Buchnera aphidicola sp. strain APS
Source: BMC Genomics. 2010 Nov 25;11:666. doi: 10.1186/1471-2164-11-666 (PMC3091783; doi:10.1186/1471-2164-11-666)
Supplement: Additional file 3 — Quality of the predictions for the three predictor models of DisTer. [file 1471-2164-11-666-S3.PDF]

Quality of the predictions for the three predictor models of DisTer

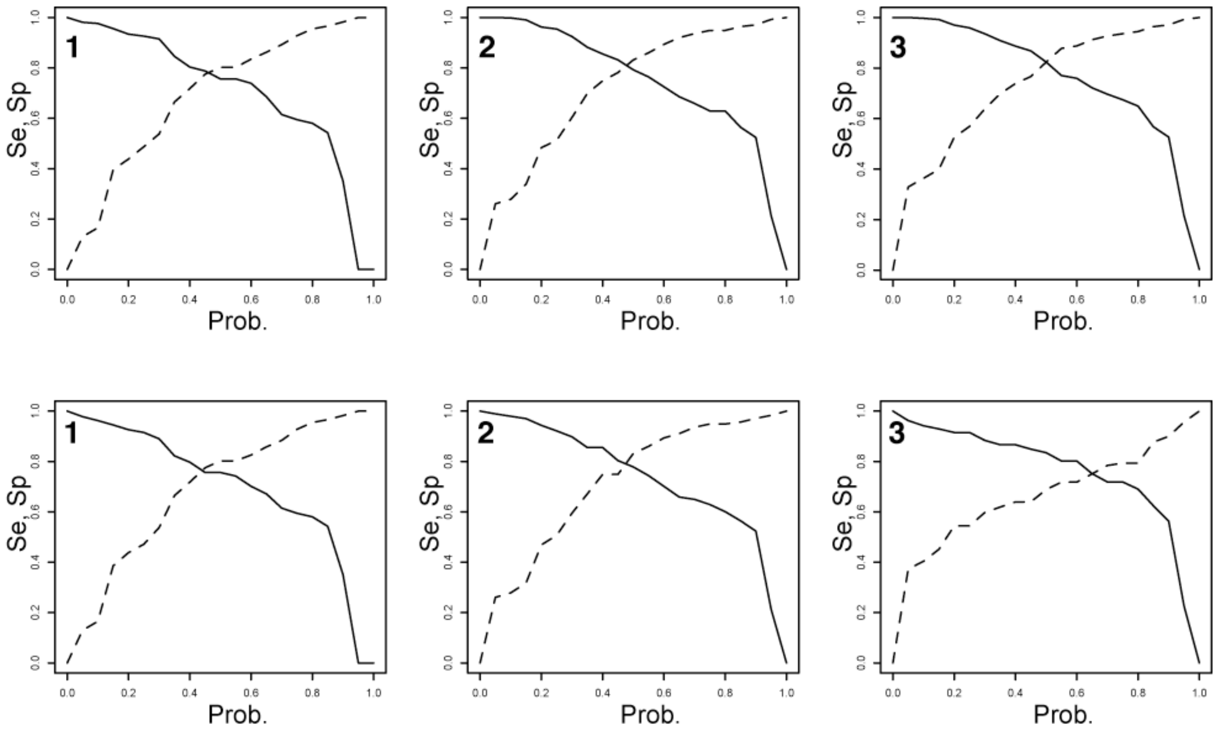

The quality of predictions made with the three models (see Materials and methods section for the models definitions) of DisTer regarding the probability threshold. For the first line of graphs, the sensitivity (Se) and specificity (Sp) was computed using the prediction of TUs for the entire *E. coli* data set, the totality of this data was used for the training. The values of Se and Sp are a good indicator of the uncertainty of the classification rules. The second line of graphs pictures the models predictive capability using the leave-one-out cross validation technique.
