# Supplementary material for: Structure and dynamics of the operon map of Buchnera aphidicola sp. strain APS
Source: BMC Genomics. 2010 Nov 25;11:666. doi: 10.1186/1471-2164-11-666 (PMC3091783; doi:10.1186/1471-2164-11-666)
Supplement: Additional file 4 — List of the B. aphidicola TUs predicted with DisTer. [file 1471-2164-11-666-S4.PDF]

List of the *B. aphidicola* TUs predicted with DisTer

TU	TU genes
1	*gidA*
2	*atpB, atpE, atpF, atpH, atpA, atpG, atpD, atpC*
3	*gyrB, dnaN, dnaA*
4	*rpmH, rnpA*
5	*yidC, thdF*
6	*tRNA-Phe-GAA*
7	*groES, mopA*
8	*efp*
9	*dnaC, dnaT, yhhF*
10	*ftsY*
11	*rpoH*
12	*glmS, glmU*
13	*yigL*
14	*metR*
15	*metE*
16	*purH*
17	*hupA*
18	*rpoC, rpoB*
19	*rplL, rplJ*
20	*rplA, rplK, nusG, secE*
21	*tRNA-Thr-GGT, tRNA-Gly-TCC, tRNA-Tyr-GTA, tRNA-Thr-TGT*
22	*murB*
23	*metF*
24	*argE*
25	*argC, argB, argG, argH, yibN*
26	*secB*
27	*cysE*
28	*rpoD*
29	*dnaG, rpsU*
30	*ygjD*
31	*ribB*
32	*rfaE*
33	*cca*
34	*uppP, crr, ptsI*
35	*ptsH*
36	*cysK*
37	*lig*
38	*tRNA-Lys-TTT, tRNA-Val-TAC*
39	*gltX*
40	*tRNA-Ala-GGC*
41	*fliE*
42	*fliF, fliG, fliH, fliI, fliJ, fliK, fliM, fliN, fliOP, fliQ, fliR*
43	*rpmG, rpmB*
44	*ytfN*
45	*ppa*
46	*pmbA*
47	*rnpB, yraL*

Structure and dynamics of the operon map of *Buchnera aphidicola* sp. strain APS

Lilia Brinza, Federica Calevro, Gabrielle Duport, Karen Gaget, Christian Gautier, Hubert Charles

**48**	*fabB*
**49**	*talA, tktB*
**50**	*dapE*
**51**	*dapA*
**52**	*aroC*
**53**	*yfcN*
**54**	*hisG, hisD, hisC, hisB, hisH, hisA, hisF, hisI*
**55**	*gnd*
**56**	*dcd*
**57**	*metG*
**58**	*mesJ, tRNA-Val-GAC*
**59**	*ribE, rnfA, rnfB*
**60**	*rnfC, rnfD*
**61**	*rnfG, ydgQ, nth, priA*
**62**	*tyrS*
**63**	*sufA*
**64**	*ydiK*
**65**	*aroH*
**66**	*thrS, infC, rpmI, rplT*
**67**	*pheS, pheT, himA, queA, tgt, yajC*
**68**	*glyS, glyQ*
**69**	*folE*
**70**	*nfo, rplY*
**71**	*yabI*
**72**	*surA, ksgA, apaH*
**73**	*folA, carB, carA*
**74**	*dapB, lytB, lspA, ileS, ribF*
**75**	*rpsT*
**76**	*dnaJ, dnaK*
**77**	*nuoA, nuoB, nuoCD, nuoE, nuoF, nuoG, nuoH, nuoI, nuoJ, nuoK, nuoL, nuoM, nuoN*
**78**	*folC, cvpA*
**79**	*prsA*
**80**	*ispE*
**81**	*prfA, hemK*
**82**	*ychA*
**83**	*nadE*
**84**	*ackA, pta*
**85**	*yfaE, nrdB, nrdA, gyrA*
**86**	*yba2*
**87**	*ahpC*
**88**	*ung*
**89**	*grpE*
**90**	*nadK*
**91**	*smpA*
**92**	*ssrA*
**93**	*ydhD*
**94**	*rnt*
**95**	*sodA*
**96**	*pth, ychF*
**97**	*thrC, thrB, thrA*
**98**	*hpt*
**99**	*panC, panB, dksA*

Structure and dynamics of the operon map of *Buchnera aphidicola* sp. strain APS

Lilia Brinza, Federica Calevro, Gabrielle Duport, Karen Gaget, Christian Gautier, Hubert Charles

100 *truA, mrcB*
101 *secA, mutT*
102 *coaE*
103 *guaC*
104 *aceE, aceF, lpdA*
105 *speD, speE*
106 *pfs, yadR*
107 *ftsZ, ftsA, ddlB*
108 *murC, murG, ftsW, murD, mraY, murF, murE, ftsI, ftsL, yabC, ilvH, ilvI*
109 *apbE*
110 *htrA*
111 *dapD, map*
112 *rpsB, tsf, pyrH, frr, dxr, uppS*
113 *yaeT, dnaE*
114 *proS*
115 *flhB, flhA*
116 *argS*
117 *rrs*
118 *tRNA-Ile-GAT, tRNA-Ala-TGC*
119 *gloB, rnhA*
120 *dnaQ, tRNA-Asp-GTC, lpcA*
121 *gpt*
122 *grpE1*
123 *yfjF*
124 *smpB, yfhC*
125 *acpS, era, rnc*
126 *lepB, lepA*
127 *trmU, ycfC, purB, mltE, fabI*
128 *rnb*
129 *ychE, lipB, lipA*
130 *pyrF, ribA*
131 *hns*
132 *cls*
133 *yciA, yciB, yciC*
134 *trpA, trpB, trpC, trpD*
135 *yedA, rluB, sohB, topA*
136 *suhB*
137 *yfgB, gcpE, hisS, glyA*
138 *bioD*
139 *bioB*
140 *bioA*
141 *pgl, mfd*
142 *lolC, lolD, lolE, gapA*
143 *fldA*
144 *phrB, ybgI, sucA, sucB, gpmA*
145 *pfkA, glpF*
146 *tpiA, himD, rpsA*
147 *cmk, aroA, serC, serS*
148 *trxB*
149 *infA*
150 *aspS*
151 *znuB, znuC*

Structure and dynamics of the operon map of *Buchnera aphidicola* sp. strain APS

Lilia Brinza, Federica Calevro, Gabrielle Duport, Karen Gaget, Christian Gautier, Hubert Charles

**152**	*pykA*
**153**	*zwf*
**154**	*htpX*
**155**	*cspC*
**156**	*yoaE, yeaZ*
**157**	*minE, minD, minC*
**158**	*rsmC*
**159**	*tRNA-Leu-TAA, tRNA-Cys-GCA*
**160**	*tRNA-Ser-TGA*
**161**	*ompA*
**162**	*mviN*
**163**	*pyrC*
**164**	*flgN, flgA*
**165**	*flgB, flgC, flgD, flgE, flgF, flgG, flgH*
**166**	*flgI, flgJ*
**167**	*flgK*
**168**	*rne*
**169**	*rluC, rpmF*
**170**	*fabD, fabG, acpP, tmk, holB, ycfH, ptsG, ycfF, ycfM*
**171**	*ompF*
**172**	*asnS*
**173**	*pncB*
**174**	*pyrD*
**175**	*ycbY, uup*
**176**	*yceA, valS, pepA*
**177**	*argF, pyrB, pyrI, yhaR*
**178**	*deaD*
**179**	*pnp*
**180**	*rpsO*
**181**	*truB, rbfA, infB, nusA*
**182**	*tRNA-Leu-GAG, secG*
**183**	*mrsA*
**184**	*hflB, ftsJ, greA*
**185**	*yrbA, murA*
**186**	*rplU, rpmA*
**187**	*yhbZ*
**188**	*rpsI, rplM*
**189**	*pheA*
**190**	*ffh*
**191**	*rpsP, rimM, trmD, rplS*
**192**	*tldD*
**193**	*aroQ*
**194**	*fis*
**195**	*rluD*
**196**	*yfiO*
**197**	*alaS*
**198**	*csrA*
**199**	*tRNA-Ser-GCT, tRNA-Arg-ACG*
**200**	*gshA*
**201**	*metK*
**202**	*endA, yggJ*
**203**	*rpiA*

204 *tRNA-Gln-TTG, tRNA-Leu-TAG, tRNA-Met-CAT1*
205 *glnS*
206 *pyrG, eno*
207 *nlpD, ispF, ispD, ftsB, cysC, cysN, cysD, cysG*
208 *cysH, cysI, cysJ*
209 *mutS*
210 *dsbA, polA*
211 *yihA*
212 *typA*
213 *gmk, ygfZ*
214 *prfB, lysS, lysA, lgt, thyA, yleA*
215 *ybeY, ybeX*
216 *leuS, holA, nadD, sirA*
217 *asd*
218 *yhgN, pgk, fba*
219 *mscS, recC, recB, recD*
220 *argA*
221 *tRNA-Met-CAT2*
222 *mltA, ribH, thiL, ribD1, ribD2, nusB*
223 *dxs, ispA, yajR*
224 *yccK*
225 *cyoE, cyoD, cyoC, cyoB, cyoA*
226 *bolA*
227 *tig*
228 *clpP, clpX*
229 *lon*
230 *ppiD*
231 *ybaX*
232 *mdl, mdlB*
233 *ffs*
234 *dnaX*
235 *ybaB*
236 *htpG, adk*
237 *tRNA-Arg-TCT*
238 *folD*
239 *cysS*
240 *ybeD*
241 *cspE*
242 *rrf*
243 *rrl*
244 *tRNA-Glu-TTC*
245 *aroE, yrdC, smg*
246 *def, fmt*
rplQ, rpoA, rpsD, rpsK, rpsM, rpmJ, secY, rplO, rpmD, rpsE, rplR, rplF, rpsH, rpsN, rplE, rplX, rplN, rpsQ, rpmC, rpl[illegible]
247 *rpsJ*
248 *tufA, fusA, rpsG, rpsL*
249 *yheL, yheM, yheN, fkpA*
250 *argD*
251 *tsgA*
252 *trpS, rpe*
253 *aroB, aroK*
254 *tRNA-Ser-GGA*
255 *deoD, deoB, prfC*

**256**	*yhgI*
**257**	*ssb*
**258**	*dnaB*
**259**	*gshB, yqgF, yggS, yggW*
**260**	*yggH*
**261**	*mutY, yggX, murI*
**262**	*sbcB*
**263**	*yeeX*
**264**	*tRNA-Asn-GTT*
**265**	*tRNA-Met-CAT3, pyrE*
**266**	*dut, cysQ, rplI, rpsR, rpsF*
**267**	*vacB, purA, hflC, hflK*
**268**	*miaA, mutL, mtlD, mtlA*
**269**	*pgi*
**270**	*orn, tRNA-Gly-GCC*
**271**	*amiB*
**272**	*rpmE*
**273**	*hslV, hslU*
**274**	*ibpA, fpr*
**275**	*poxA*
**276**	*kdtB*
**277**	*yba3, yba4*
**278**	*yhiQ*
**279**	*pitA*
**280**	*ynfM, dapF*
**281**	*cyaY, hemC*
**282**	*hemD*
**283**	*tRNA-Pro-TGG, tRNA-His-GTG, tRNA-Arg-CCG*
**284**	*rho, trxA*
**285**	*rep, ilvC, ilvD*
**286**	*tRNA-Trp-CCA*
**287**	*iscS, iscU, hscB, hscA, fdx*
**288**	*der, yfgM*
