# Supplementary material for: Structure and dynamics of the operon map of Buchnera aphidicola sp. strain APS
Source: BMC Genomics. 2010 Nov 25;11:666. doi: 10.1186/1471-2164-11-666 (PMC3091783; doi:10.1186/1471-2164-11-666)
Supplement: Additional file 5 — Pairs of adjacent genes for which the STU status was experimentally validated by RT-PCR. [file 1471-2164-11-666-S5.PDF]

Pairs of adjacent genes for which the STU status was experimentally validated by RT-PCR

Pairs of adjacent genes for which the STU status was experimentally validated by RT-PCR. The gene pair type indicated in the forth column is associated as following: (a) gene pairs predicted as STU solely by DisTer; (b) gene pairs predicted as STU by DisTer with no consensual annotation given by the 3 other methods; (c) gene pairs predicted as STU by all methods; (PC) positive controls.

Gene1	Gene2	Gene pair type	Interg. Dist	Ter	BioCyc	DOOR	DisTer	Microbes Online
atpB	atpE	c	37	+	STU	STU	STU	STU
atpE	atpF	b	119	+	DTU	DTU	STU	STU
atpA	atpG	c	33	-	STU	STU	STU	STU
atpG	atpD	c	24	-	STU	STU	STU	STU
atpD	atpC	c	27	-	STU	STU	STU	STU
tsf	pyrH	b	51	+	DTU	STU	STU	STU
argC	argB	c	21	+	STU	STU	STU	STU
argB	argG	c	30	+	STU	STU	STU	STU
argG	argH	b	72	-	STU	DTU	STU	STU
holA	nadD	c	23	-	STU	STU	STU	STU
trpA	trpB	PC	19	-	STU	STU	STU	STU
trpB	trpC	PC	38	-	STU	STU	STU	STU
rho	trxA	a	130	+	DTU	DTU	STU	DTU
ycfH	ptsG	a	97	+	DTU	DTU	STU	DTU
secA	mutT	a	76	+	DTU	DTU	STU	DTU
rnpB	yraL	a	80	+	DTU	DTU	STU	DTU
nrdA	gyrA	a	73	+	DTU	DTU	STU	DTU
lysA	lgt	a	66	-	DTU	DTU	STU	DTU
tkyA	yleA	a	67	-	DTU	DTU	STU	DTU
queA	tgt	c	41	-	STU	STU	STU	STU
ybeY	ybeX	b	81	+	DTU	DTU	STU	STU
nuoI	nuoJ	c	10	+	STU	STU	STU	STU
yceA	valS	a	57	-	DTU	DTU	STU	DTU
secE	tRNA-Thr	d	314	-	DTU	DTU	DTU	DTU
dapA	aroC	d	576	+	DTU	DTU	DTU	DTU
cls	yciA	d	381	+	DTU	DTU	DTU	DTU
fkpA	argD	d	463	+	DTU	DTU	DTU	DTU
hisI	gnd	d	359	+	DTU	DTU	DTU	DTU
dnaB	gshB	d	247	+	DTU	DTU	DTU	DTU
mrsA	hflB	d	220	+	DTU	DTU	DTU	DTU
dnaX	ybaB	d	320	+	DTU	DTU	DTU	STU

Structure and dynamics of the operon map of *Buchnera aphidicola* sp. strain APS

Lilia Brinza, Federica Calevro, Gabrielle Duport, Karen Gaget, Christian Gautier, Hubert Charles
