# Supplementary material for: Structure and dynamics of the operon map of Buchnera aphidicola sp. strain APS
Source: BMC Genomics. 2010 Nov 25;11:666. doi: 10.1186/1471-2164-11-666 (PMC3091783; doi:10.1186/1471-2164-11-666)
Supplement: Additional file 6 — Experimental validation by RT-PCR for 4 complete operons. [file 1471-2164-11-666-S6.PDF]

Experimental validation by RT-PCR for 4 complete operons

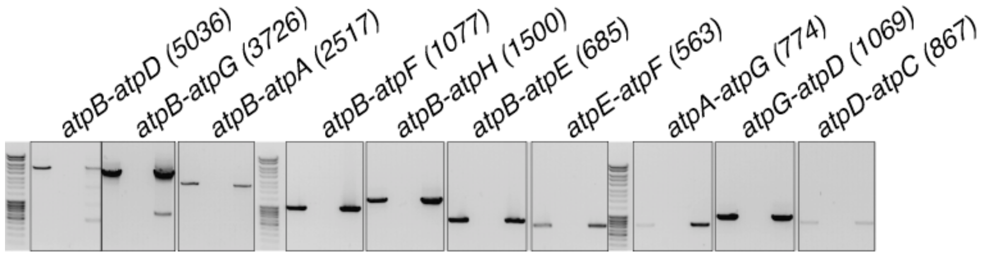

Experimental validation by RT-PCR for the complete *atpBEFHAGDC* operon**.** For each pair of primers there was a negative control, to which the RT enzyme was not added (the second column), and a positive control, in which we used gDNA instead the cDNA (the third column). The size (bp) of the amplicon is indicated next to the amplicon name.

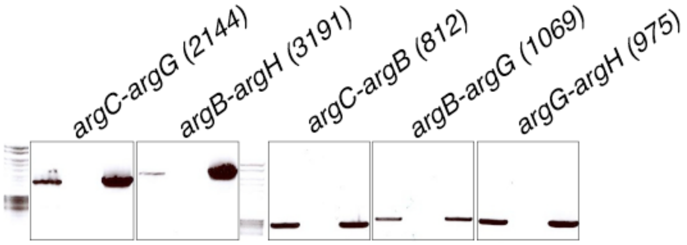

Experimental validation by RT-PCR for the complete *argCBGH* operon**.** For each pair of primers there was a negative control, to which the RT enzyme was not added (the second column), and a positive control, in which we used gDNA instead the cDNA (the third column). The size (bp) of the amplicon is indicated next to the amplicon name.

Structure and dynamics of the operon map of *Buchnera aphidicola* sp. strain APS

Lilia Brinza, Federica Calevro, Gabrielle Duport, Karen Gaget, Christian Gautier, Hubert Charles

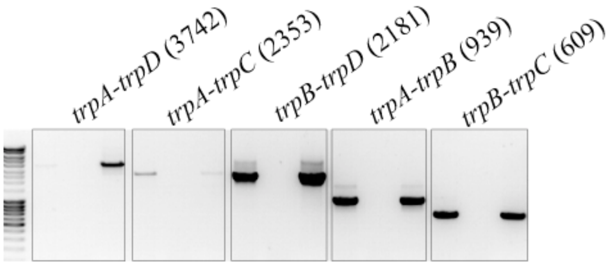

Experimental validation by RT-PCR for the complete *trpABCD* operon. For each pair of primers there was a negative control, to which the RT enzyme was not added (the second column), and a positive control, in which we used gDNA instead the cDNA (the third column). The size (bp) of the amplicon is indicated next to the amplicon name.

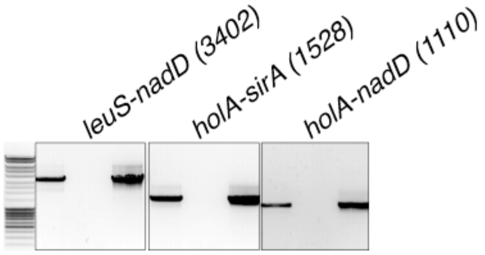

Experimental validation by RT-PCR for the complete *leuSholAnadDsirA* operon**.** For each pair of primers there was a negative control, to which the RT enzyme was not added (the second column), and a positive control, in which we used gDNA instead the cDNA (the third column). The size (bp) of the amplicon is indicated next to the amplicon name.

Structure and dynamics of the operon map of *Buchnera aphidicola* sp. strain APS

Lilia Brinza, Federica Calevro, Gabrielle Duport, Karen Gaget, Christian Gautier, Hubert Charles
