# Supplementary material for: Structure and dynamics of the operon map of Buchnera aphidicola sp. strain APS
Source: BMC Genomics. 2010 Nov 25;11:666. doi: 10.1186/1471-2164-11-666 (PMC3091783; doi:10.1186/1471-2164-11-666)
Supplement: Additional file 8 — Buchnera vs. E. coli gene pair status comparison. [file 1471-2164-11-666-S8.PDF]

Buchnera vs. *E. coli* gene pair status comparison

	Same strand pairs		Opposite strand pairs	
	443		167	
	STU pairs	DTU pairs	Convergent DTU pairs	Divergent DTU pairs
	323	120	84	83
The orthologous gene-pairs in *E. coli* are adjacent and belong to the same TU	188	13	-	-
The orthologous gene-pairs in *E. coli* are adjacent and belong to different TUs	21	15	3	14
The orthologous gene-pairs in *E. coli* are not adjacent but do belong to the same TU	18	6	-	-
The orthologous gene-pairs in *E. coli* are not adjacent and belong to different TUs	96	86	81	69
