# Supplementary material for: Structure and dynamics of the operon map of Buchnera aphidicola sp. strain APS
Source: BMC Genomics. 2010 Nov 25;11:666. doi: 10.1186/1471-2164-11-666 (PMC3091783; doi:10.1186/1471-2164-11-666)
Supplement: Additional file 9 — Intergenic distance characteristics for each pair type. [file 1471-2164-11-666-S9.PDF]

Intergenic distance characteristics for each pair type

	Same strand pairs		Opposite strand pairs	
	STU pairs	DTU pairs	Convergent DTU pairs	Divergent DTU pairs
Median and mean of *Buchnera* intergenic distances	29.5, 40.7	162, 216.1	65.5, 78.5	220, 236.9
Median and mean of *E. coli* intergenic distances	10, 23.96	134, 158.8	52, 87.4	208, 234.5
Wilcoxon test p-value (*Buchnera* intergenic distances vs. *E. coli* intergenic distances)	< 2.2e-16 ***	0.001*	0.81	0.57

Structure and dynamics of the operon map of *Buchnera aphidicola* sp. strain APS

Lilia Brinza, Federica Calevro, Gabrielle Duport, Karen Gaget, Christian Gautier, Hubert Charles
