# Supplementary material for: Structure and dynamics of the operon map of Buchnera aphidicola sp. strain APS
Source: BMC Genomics. 2010 Nov 25;11:666. doi: 10.1186/1471-2164-11-666 (PMC3091783; doi:10.1186/1471-2164-11-666)
Supplement: Additional file 10 — Distributions of the scores of the predicted σ70 promoters. [file 1471-2164-11-666-S10.PDF]

Distributions of the scores of the predicted σ^{70} promoters

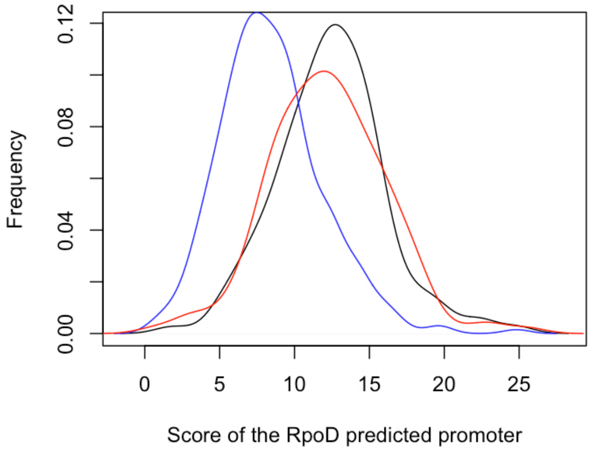

The distribution of the scores of the predicted σ^{70} promoters upstream first genes of operons (black), monocistronic TU genes (red) and inner genes of operon (blue).

Structure and dynamics of the operon map of *Buchnera aphidicola* sp. strain APS

Lilia Brinza, Federica Calevro, Gabrielle Duport, Karen Gaget, Christian Gautier, Hubert Charles
