# Supplementary material for: Structure and dynamics of the operon map of Buchnera aphidicola sp. strain APS
Source: BMC Genomics. 2010 Nov 25;11:666. doi: 10.1186/1471-2164-11-666 (PMC3091783; doi:10.1186/1471-2164-11-666)
Supplement: Additional file 11 — Buchnera gene Ka distributions. [file 1471-2164-11-666-S11.PDF]

Buchnera gene Ka distributions

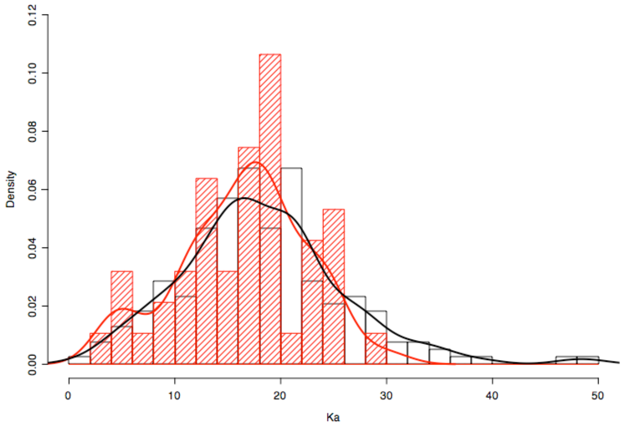

Buchnera gene Ka distributions. In black (red) is depicted the distribution of Kas of genes shorter (longer) than their orthologues and which have shrank (extended) in the *Buchnera* lineage as compared to that of *E. coli*. The two distributions are similar (Wilcoxon test, p-value= 0.27).

Structure and dynamics of the operon map of *Buchnera aphidicola* sp. strain APS

Lilia Brinza, Federica Calevro, Gabrielle Duport, Karen Gaget, Christian Gautier, Hubert Charles
