# Supplementary material for: Structure and dynamics of the operon map of Buchnera aphidicola sp. strain APS
Source: BMC Genomics. 2010 Nov 25;11:666. doi: 10.1186/1471-2164-11-666 (PMC3091783; doi:10.1186/1471-2164-11-666)
Supplement: Additional file 12 — Distribution of E. coli TUs in the Buchnera TU classes. [file 1471-2164-11-666-S12.PDF]

Distribution of *E. coli* TUs in the *Buchnera* TU classes

Distribution of *E. coli* TUs (including at least one gene for which an orthologue was found in the *Buchnera* genome) in the *Buchnera* TU classes. The Chi-test p-value was 0.04 when all classes were included, but this significant result was attributable to the Split class, for which there were very low counts; the Chi-2 test performed without the Split class was not significant (p-value = 0.3).

	Identical	Similar	Split	Merged	Reorganized
Regulated TU in *E. coli*	48	14	10	49	12
Not regulated TU in *E. coli*	72	40	5	101	21

Structure and dynamics of the operon map of *Buchnera aphidicola* sp. strain APS

Lilia Brinza, Federica Calevro, Gabrielle Duport, Karen Gaget, Christian Gautier, Hubert Charles
