# Supplementary material for: Structure and dynamics of the operon map of Buchnera aphidicola sp. strain APS
Source: BMC Genomics. 2010 Nov 25;11:666. doi: 10.1186/1471-2164-11-666 (PMC3091783; doi:10.1186/1471-2164-11-666)
Supplement: Additional file 13 — Counts of ancestral genes conserved or not conserved in Buchnera depending on their regulation in E. coli. [file 1471-2164-11-666-S13.PDF]

Counts of ancestral genes conserved or not conserved in *Buchnera* depending on their regulation in *E. coli*

Counts of ancestral genes conserved or not conserved in *Buchnera* depending on whether they are specifically regulated in *E. coli* or not. The set of genes of the last common ancestor of *E. coli* and *Buchnera* was constructed using the same method as that described in the Methods section for the Coding sequence length evolution. Chi-test p-value = 0.008.

	Conserved in *Buchnera*	Not conserved in *Buchnera*
Regulated in *E. coli*	247	831
No regulated in *E. coli*	344	1488
